# West Texas Medieal Association

**Published:** 1902-01

**Authors:** W. B. Russ

**Affiliations:** Secretary


### West Texas Medical Association.

The West Texas Medical Association held an unusually interesting meeting in San Antonio on Thursday, December 5th. Among the members present were Drs. P. Baldarsarelli, James Bartlett, E. C. Clavin, C. M. Decker, R. L. Dinwiddie, F. M. Hicks, E. F. Hughes, T. T. Jackson, C. E. R. King, B. F. Kingsley, W E. Luter, A. S. McDaniel, Robert E. Moss, T. E. Moor, F. Paschal, R. A. Goeth, E. H. Elmendorf, W. B. Russ, Willis P. King, C. Warfield, J. P. Rote, C. R. Newrick, and Fred Terrell, of city council.

An excellently prepared paper on "The Wounds of War" was read by Dr. T. T. Jackson, who has seen active service in the Philippine Islands while serving as assistant surgeon in the U. S. army. The discussion was led by Dr. James Bartlett. Following Dr. Bartlett came Drs. Willis P. King, F. M. Hicks and A. S. McDaniel.

Cases for clinical study were presented by Drs. F. Paschal and B. F. Kingsley, and interesting reports of cases were made by Drs. A. S. McDaniel, Robert E. Moss and C. E. R. King.

A resolution to endorse the proposed city ordinance to regulate the undertaking establishments operating within the city limits was unanimously adopted. The provisions of the ordinance were fully outlined and explained by Drs. Paschal and Terrell.

During the past two months the West Texas Medical Association has increased its membership from fifty-six to seventy-two, and there are at present ten or more applications under consideration.

The program for every regular meeting of the society now includes (1) a paper, (2) discussion on the paper, the leader having been appointed several days prior to the date of meeting; (3) presentation of cases for clinical study—this has proved to be a most interesting feature, (4) reports of cases, followed by discussion; (5) pathological specimens.

The association is now in a more prosperous condition than it has ever been before during its twenty-five years of existence.

W. B. Russ, M. D., Secretary.

